# Pathogenic Role of Immune Evasion and Integration of Human Papillomavirus in Oropharyngeal Cancer

**DOI:** 10.3390/microorganisms9050891

**Published:** 2021-04-21

**Authors:** Takashi Hatano, Daisuke Sano, Hideaki Takahashi, Nobuhiko Oridate

**Affiliations:** Department of Otorhinolaryngology, Head and Neck Surgery, School of Medicine, Yokohama City University, 3-9 Fukuura, Kanazawa-Ku, Yokohama 236-0004, Japan; dsano@yokohama-cu.ac.jp (D.S.); htk98@yokohama-cu.ac.jp (H.T.); noridate@yokohama-cu.ac.jp (N.O.)

**Keywords:** human papillomavirus, HPV, HPV-induced oncogenesis, oropharyngeal cancer, immune evasion, viral integration

## Abstract

The incidence of oropharyngeal cancer (OPC) is increasing remarkably among all head and neck cancers, mainly due to its association with the human papillomavirus (HPV). Most HPVs are eliminated by the host’s immune system; however, because HPV has developed an effective immune evasion mechanism to complete its replication cycle, a small number of HPVs are not eliminated, leading to persistent infection. Moreover, during the oncogenic process, the extrachromosomal HPV genome often becomes integrated into the host genome. Integration involves the induction and high expression of E6 and E7, leading to cell cycle activation and increased genomic instability in the host. Therefore, integration is an important event in oncogenesis, although the associated mechanism remains unclear, especially in HPV-OPC. In this review, we summarize the current knowledge on HPV-mediated carcinogenesis, with special emphasis on immune evasion and integration mechanisms, which are crucial for oncogenesis.

## 1. Introduction

Head and neck cancer (HNC) is the sixth most common cancer, accounting for approximately 3–5% of all cancers, with more than 500,000 diagnoses worldwide every year [1,2]. The leading causes of head and neck squamous cell carcinoma (HNSCC) are smoking and alcohol consumption and viral infections have recently attracted attention as a third factor. In particular, the incidence of human papillomavirus (HPV)-associated oropharyngeal cancer (HPV-OPC) has been on the rise [3]. In the United States, it has been reported that the incidence of HPV-OPC is likely to exceed that of cervical cancer, which is also an HPV-associated disease [4].

HPV-induced carcinogenesis was first highlighted by the detection of HPV16 in cervical cancer tissues by Zur Hausen et al. in 1983 [5]. Syrjanen et al. first reported a relationship between HPV infection and oral cancer [6]. In cervical cancer, HPV16 and HPV18 are the main HPV types [7], whereas more than 90% of HPV-OPCs are caused by HPV16 infection [8]. HPV-OPC patients have several unique characteristics, including younger age, better response rates to treatment, and better prognosis than smoking and alcohol consumption-related HNSCC [8,9]. Thus, HPV infection status is considered an independent prognostic factor for survival in patients with OPC [3]. For these reasons, in the 8th edition of the American Joint Committee on Cancer/Union for International Cancer Control TNM staging system, OPC is divided according to HPV infection status [10,11]. HPV-OPC mainly occurs in the palatine and lingual tonsils, and it is considered that within 1–2 years of the initial infection, most HPVs are eliminated by the host immune system. However, a small number of HPVs are not eliminated, leading to a persistent infection that eventually develops into HPV-associated carcinoma [12]. In this regard, HPV has likely developed effective immune evasion mechanisms to complete its replication cycle. Thus, elucidating the details of the immune evasion mechanisms of HPV is important for controlling persistent infections.

Moreover, during the oncogenic process, the episomal HPV genome is often integrated into the host genome, disrupting E2 expression. The integration of the HPV genome is the most important factor associated with the stable and high expression of the two viral oncoproteins, E6 and E7. The E6 protein inhibits apoptosis by binding to ubiquitin ligase, thereby degrading the p53 protein and immortalizing cells by increasing telomerase activity. The E7 protein activates E2F by binding to pRb, leading to cell cycle activation and increased genomic instability in the host [7]. Therefore, HPV genome integration is a crucial event in oncogenesis, although the associated mechanism remains unclear, especially in HPV-OPC. This review summarizes recent studies on the pathogenic role of HPV-driven carcinogenesis in HNSCC, highlighting HPV-mediated immune evasion systems and viral integration mechanisms.

## 2. The Genomic Structure of HPV and Viral Gene Products

**Figure 1:**
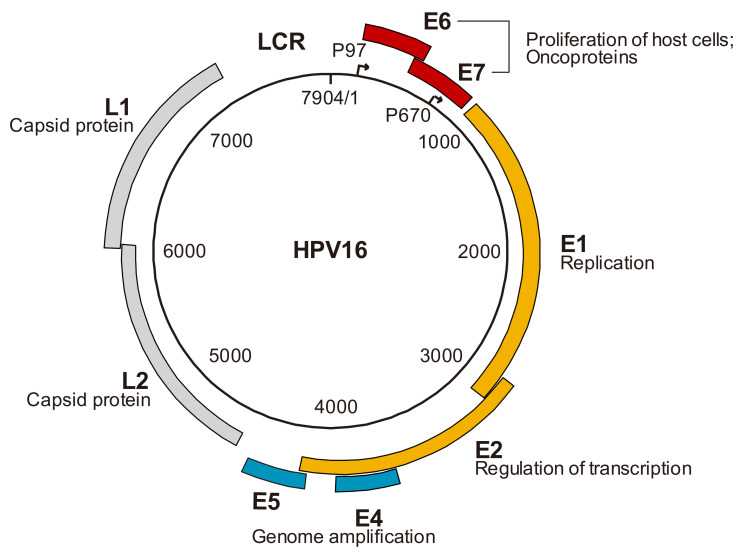
Genome organization of human papillomavirus (HPV) 16 and function of viral gene products. Schematic representation of the HPV16 genome shows location of early (E) and late (L) genes, and of the long control region (LCR).

HPV is classified as a member of the Papillomaviridae family, with non-enveloped, circular, double-stranded DNA within a spherical capsid. Currently, more than 200 types of papillomaviruses have been reported in humans, according to the HPV database [13,14]. HPV types are generally classified based on the nucleotide sequence homology of the L1 gene into five groups: alpha, beta, gamma, mu, and nu [15]. Approximately 30 HPV types, regarded as *Alphapapillomavirus* spp., infect both cutaneous epithelium and mucosa of the genital and oral tract, while other groups infect the cutaneous epithelium. Thus, most *Alphapapillomavirus* spp. are grouped as “mucosal HPV types” [16]. Mucosal HPV types can be further divided into high-risk (HR) and low-risk (LR) groups based on their carcinogenic ability [17]. Approximately 15 types, including types 16, 18, 31, 33, 35, 39, 45, 51, 52, 56, 58, 59, 68, 73, and 82, are considered HR-HPV types [18]. Conversely, LR-HPV types 6, 11, 40, 42, 43, and 44 are found primarily in benign genital warts and other non-malignant lesions, such as oral focal epithelial hyperplasia [16]. Interestingly, although the classification of the HR and LR groups was originally based on carcinogenic risk, HR types are concentrated in specific subgroups on the molecular phylogenetic tree, suggesting that epidemiologic and phylogenetic classifications of HPV types are correlated [18,19]. The HPV genome consists of approximately 7900 base pairs and contains early (E1, E2, E4, E5, E6, and E7) and late (L1 and L2) genes, as well as a long control region (LCR) as shown in the schematic representation of the HPV16 genome in Figure 1.

The early genes are expressed during the early stages of viral replication, the late genes encode the major (L1) and minor (L2) capsid proteins, and the LCR, localized between open reading frames (ORFs) L1 and E6, encodes the viral genome replication origin and binding sites for various transcription factors involved in viral gene expression. Promoters are located upstream of the E6 ORF and in the E7 ORF, which are thought to regulate early and late gene expression, respectively. Early genes, except for E4, are expressed in undifferentiated basal cells, while expression of E4 and late genes is induced in response to the differentiation of infected cells [20]. The functions of the early and late genes are as follows (Figure 1): E1 is a helicase that recruits the cellular factors of the host to replicate the HPV genome; E2 has multiple binding sites (BS) in the LCR and regulates viral transcription, and also has the function of recruiting E1 to the replication origin by constructing a heterodimer complex (during viral replication); E4 has the function of HPV genome amplification through modifying the cellular environment; E5 also has the function of HPV genome amplification through the ability to stabilize EGFR and to enhance EGF signaling; E6 and E7 play a role in oncoprotein that acts through the blocking of normal host cell division; and L1 and L2 are capsid proteins that form icosahedral capsids [7,20,21,22].

## 3. HPV Viral Life Cycle

**Figure 2:**
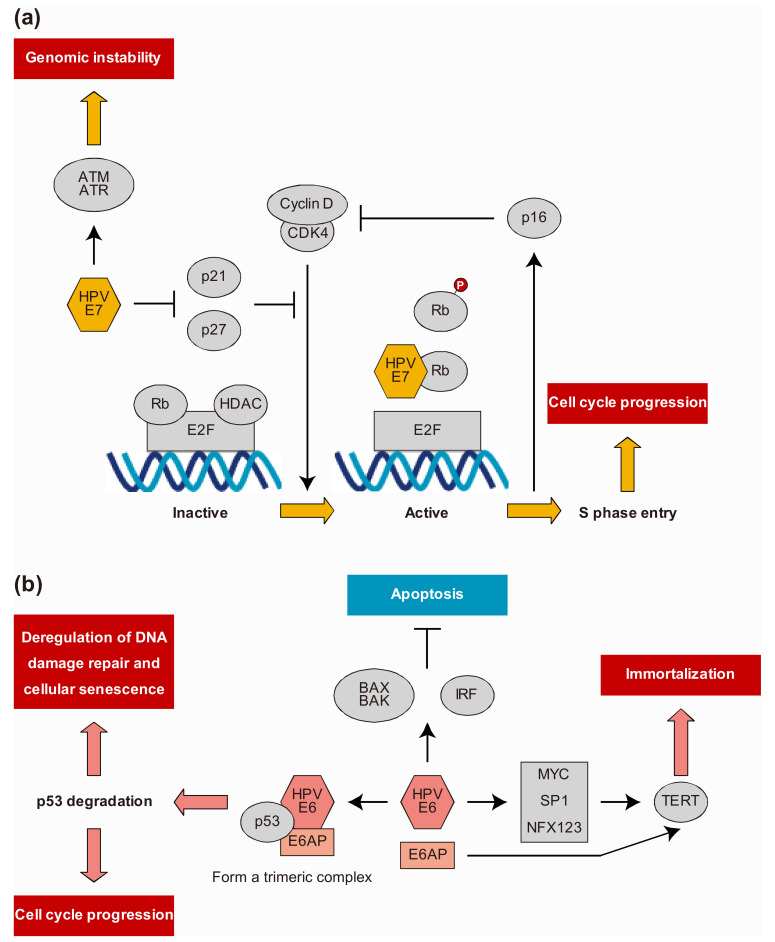
(**a**) Role of human papillomavirus (HPV) E7 oncoprotein in the progression to cancer. HR HPV E7 protein targets retinoblastoma (Rb) family members for degradation, resulting in the release and activation of E2F transcription factors that drive the expression of S phase genes as well as induction of hyperproliferation. E7 can also induce cellular proliferation through deregulation of cyclin-dependent kinase (CDK) inhibitors p21 and p27, leading to direct activation of CDK2. Degradation of Rb family members by HPV E7 protein leads to p16 overexpression, inhibiting the ability of CDK4 to interact with cyclin D and stimulating passage through the G_1_ phase of the cell cycle. (**b**) Role of HPV E6 oncoprotein in the progression to cancer. HR-HPV E6 inhibits p53-dependent growth arrest, resulting in the induction of genomic instability and the accumulation of cellular mutations. HR HPV E6 protein binds to cellular ubiquitin ligase E6AP to form a trimeric complex with p53, leading to the ubiquitylation and proteasomal degradation of p53 and the inhibition of p21. E6 also activates telomerase reverse transcriptase (TERT) and telomerase, resulting in cell proliferation and promoting immortalization.

The HPV viral life cycle is closely related to the differentiation of host epithelial cells [16]. HPV first infects undifferentiated cells in the basal layer of the epithelium, probably through micro-wounds on the epithelial surface. It exploits the lateral elongation of basal cells during wound healing to gain entry into the cell [12]. HPV undergoes initial replication and becomes an extrachromosomal episome of approximately 50–200 copies in the nucleus, resulting in latent and persistent infection without producing viral particles. At the early stage, viral gene expression is tightly controlled at a low level by E2 [16,23]. The viral genome maintains a constant copy number by maintenance replication, that is, it is replicated approximately once during the DNA synthesis phase (S phase) of infected cells and distributed to daughter cells during cell division [20]. Replication takes place only in the suprabasal, differentiating cells destined for maturation and senescence, and therefore does not naturally express the replication machinery that the virus needs for survival [24]. HPV resolves this problem by encoding two proteins, E6 and E7. The E7 protein targets and degrades retinoblastoma (Rb) family members consisting of p105 (Rb), p107, and p130, resulting in the release and activation of the E2F transcription factor, which promotes S phase gene expression and induces hyperproliferation [7]. E7 can also induce immortalization by inhibiting cyclin-dependent kinase (CDK) inhibitors p21 and p27, which are important regulators of growth arrest during epithelial differentiation. The principal target of p21 and p27 in human keratinocytes is CDK2, which is vital for G1 to S phase entry; thus, inhibition of p21 and p27 leads to the activation of CDK2. Consequently, the HR E6 protein inhibits p53 through several mechanisms. The E6 protein recruits and binds to the cellular E3 ubiquitin ligase E6-associated protein to form a trimeric complex with p53, leading to ubiquitination and proteasomal degradation of p53. The E6 protein can also block transcription by binding directly to p53 and inhibiting its DNA-binding activity. Thus, the expression of E6 and E7 disrupts the regulation of the cell cycle and allows amplification of the viral genome in the cell (Figure 2) [7]. The HPV genome was amplified to more than 1000 copies per cell during the growth phase. At this stage, the capsid proteins L1 and L2 are expressed, and eventually viral particles are produced and released from the most superficial layer of the epithelium [7,12,25].

## 4. Immune Evasion Mechanisms

Viral antigens are detected only in the epithelial cells of the surface layer, away from immunological surveillance. In addition, there is no cell death, necrosis, or viremia that triggers an inflammatory response. These may be strategies by which HPV evades elimination by the host’s immune mechanisms to be undetected for long periods [26]. Furthermore, the physiological immunosuppressive mechanisms are also involved in the immune evasion mechanism of HPV-OPCs. The oropharyngeal region, including lymphoepithelial organs, is the first line of defense against foreign substances. Moreover, tissues that come into contact with non-self-antigens have well-developed mechanisms to suppress excessive immune responses and autoimmunity, and maintain the balance between immunity and tolerance. Programmed death-ligand 1, which is deeply involved in immune escape mechanisms, is highly expressed, not on the surface epithelium of tonsils but on the reticulated epithelium of the deep crypts, which is the leading origin site of HPV-OPC [27]. In addition to the mechanism mentioned above, natural immune control against HPV infection is mediated by innate and acquired immunity [28].

### 4.1. Innate Immune System

The innate immune response is the first line of host defense against pathogens, including HPV. To detect and respond to pathogens, most cells of the innate immune system express pathogen recognition receptors (PRRs), such as toll-like receptors (TLRs), NOD-like receptors (NLRs), and RIG-I-like receptors (RLRs) [29,30,31,32,33,34,35,36]. TLR9 acts as a sensor by recognizing unmethylated double-stranded DNA CpG motifs present in the genome of viruses, such as HPVs [37]. Hasan et al. reported that HPV16 E7 downregulates TLR9 in human epithelial cells by recruiting histone demethylase JARID1B and histone deacetylase 1 (HDAC1) to specific regions of the TLR9 promoter and suppresses interferon response [38,39]. Furthermore, the cyclic guanosine monophosphate-adenosine monophosphate synthase/stimulator of interferon genes (cGAS/STING) pathway has been identified as an important defense mechanism against DNA viruses. cGAS is a cytosolic DNA sensor that activates type I interferon activity in a STING-dependent manner [40,41]. Lau et al. reported HPV18 E7-mediated inhibition of the cGAS/STING pathway in HeLa cells, a cervical carcinoma-derived cell line in which HPV18 DNA has been integrated [42]. However, E7 from HPV16, the dominant subtype of HPV-HNSCC, showed low homology to HPV18 E7. Luo et al. proposed that HPV16 may have a different immune evasion mechanism from that of HPV18 and showed that HPV16 E7 interacts with NLRX1 and promotes autophagy-dependent STING degradation [43]. Recently, Medler et al. reported that the complement system, an essential component of innate immunity, is associated with carcinogenesis. Using an HPV-infected mouse model, they showed that urokinase secreted by macrophages cleaved the fifth component of complement (C5) to produce C5a (an activated form of C5) and that the expression of the C5a receptor (C5aR1) was strongly induced in leukocytes. Furthermore, they reported that the produced C5a stimulated C5aR1 to produce chronic inflammation and that the C5a-mediated signal suppressed the infiltration of CD8+ T cells into the tissues [44]. It has been reported that binding of C5 to C5aR1 activates NLRP3 molecular complex formation through reactive oxygen species (ROS) and promotes inflammasome formation [45]. These mechanisms may also be associated with the process by which HPV evades the immune system.

### 4.2. Antigen Presentation

Antigen-presenting cells (APCs), such as dendritic cells (DCs) and macrophages, are immune cells that play important roles in activating T-cell-mediated immunity. APCs constitute an efficient bridge that connects the innate immune response to the adaptive immune system. However, HPVs inhibit the function of DCs through different strategies. The frequency and distribution of Langerhans cells (LCs), which are epidermal DCs present at the site of HPV infection, change with disease progression [46]. One of the possible mechanisms is that HPV downregulates NF-κB signaling in HR HPV-infected keratinocytes (KCs), inhibiting KC-derived CCL20 expression, which affects LCs [47,48], and thus limiting the capacity of LCs to stimulate cytotoxic CD8+ T cells [49]. Moreover, immunohistochemical analysis showed that reduced CCL20 levels in HPV-HNSCC patients correlated with a significant decrease in LC infiltration into tumors [50].

### 4.3. Adaptive Immune System

Cell-mediated immunity, by both T-helper (Th) cells and cytotoxic T cells (CTL, CD8+), plays a critical role in the elimination of HPV [51,52]. It has been reported that in HPV-OPSCCs, higher numbers of B cells and CD8+ T cells infiltrating tumors positively correlate with the response to immune-checkpoint blockade [53] and are associated with better clinical outcomes [54]. There are two types of Th responses: Th1 and Th2. Th1 responses are usually directed against intracellular pathogens, producing proinflammatory cytokines (IFN-γ, IL-1β, IL-2, and IL-18), whereas Th2 responses are usually induced in infections with extracellular pathogens, producing IL-4, IL-5, IL-6, IL-10, and IL-13 [55]. HR HPVs have the ability to shift the immune response from Th1 to Th2 during early HPV-induced lesions, and this shift may perturb the proper immune response, thus promoting disease progression [56]. Considering HPV carcinogenesis, the perspective of the immune microenvironment is important. In fact, HPV-OPCs are thought to have a more specific immune microenvironment than HPV-negative HNSCC [57]. However, in HPV-OPC, some reports indicate wide variations in immunologic characteristics among cases [58,59]. Kim et al. classified the immune microenvironment of HPV-associated oropharyngeal carcinoma according to the degree of CD8+ T-cell infiltration, and they reported that immune-rich types responded more favorably to immune-checkpoint blockade [60].

## 5. HPV Integration Analysis

In the course of a long, persistent infection, the extrachromosomal HPV genome is often integrated into the host genome, which is another crucial event in HPV oncogenesis. Integration usually causes E2 dysregulation and upregulation of E6 and E7 expression. Dysregulation of E6 and E7 regulates the cell cycle so effectively that infected cells never mature, promote cell proliferation, and abrogate cell cycle checkpoints. Consequently, genomic instability leads to the accumulation of genetic mutations, which eventually lead to cancer [7,9,61]. While integration occurs frequently in HPV-associated cancers, it is not a universal event. Among HPV-associated cancers, HPV exists in a variety of states: extrachromosomal viral genomes, integrated HPV genomes, or mixed [62]. In cervical cancer, HPV integration can be detected in premalignant lesions, and the integration rate increases with progression to invasive cancer [63], and the state of integrated HPV correlates with poor patient performance in comparison to the extrachromosomal state [64]. In HPV-HNC, as well as in cervical cancer, the integrated state of HPV showed poor clinical outcomes [65]. To date, the most widely reported method for detecting HPV integration sites is the polymerase chain-reaction-based approach. However, HPV DNA or RNA amplification methods are limited by technical biases [66]. Progress in the HPV integration mechanism study has been largely supported by advances in sequencing technology using next-generation sequencing (NGS).

### 5.1. HPV Genomic Integration Sites in the Host

**Figure 3:**
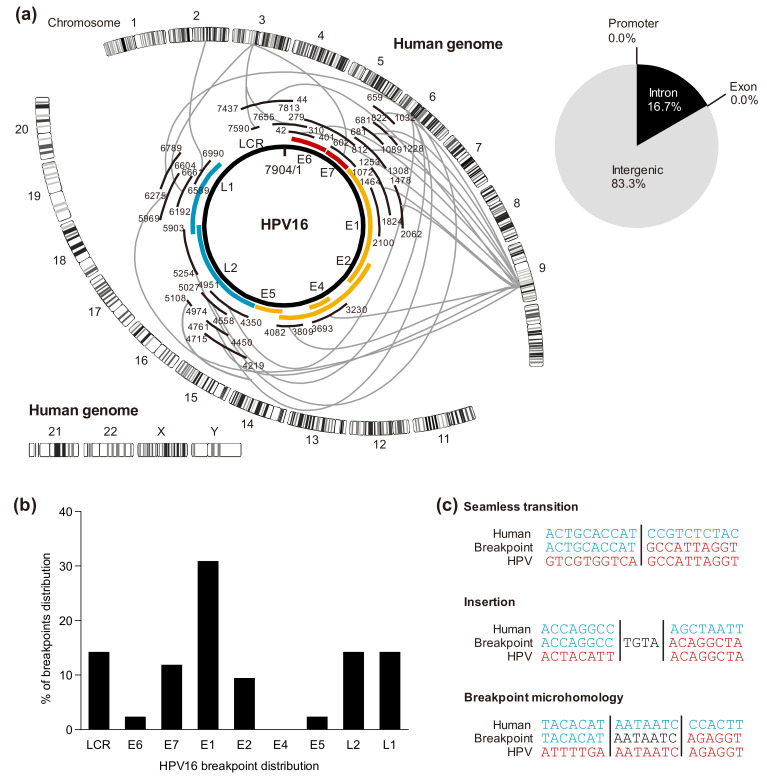
(**a**) Integration breakpoint distribution in human chromosomes and HPV16 genome represented by Circos plots of the UCSC:SCC090 genome. (**b**) Breakpoint distribution percentage in HPV16 cell lines (UCSC:SCC090, UCSC:SCC152, UCSC:SCC154). (**c**) Categories of viral-host breakpoints; (1) Seamless transition with defined breakpoint. (2) Insertion; nucleotides that align to neither reference genome. (3) Breakpoint microhomology; nucleotides that align to both reference genomes.

We previously analyzed integration sites in HPV-HNSCC cell lines using NGS with hybridizing HPV probes and detected multiple integration sites (Figure 3a) [67]. Several studies have also used NGS to identify HPV genomic integration sites in HPV-HNSCC [68,69,70,71,72,73]. It appears that there is no consensus sequence, and HPV can be integrated into any location throughout the human genome. However, some integration sites are recurrently observed; thus, these integration sites are called genomic “hotspots”. These “hotspots” are correlated with common fragile sites (CFSs), which are prone to break in response to DNA replication stress [73,74,75], transcriptionally active regions [71], and open chromatin marks [76]. Furthermore, in a previous study, we confirmed that these integration breakpoints are not only at or near the CFSs but are also at rare fragile sites [67]. In cervical cancers with HPV18 integration, there is a greater likelihood of integration to chromosome 8q24.21, which is near the cMYC locus, than those with HPV16 integration [71,77]; however, this is considered not to be a universal event associated with HPV integration [62,78].

### 5.2. Cleavage Sites in the HPV Genome

Previous studies have proposed that in HPV-HNSCCs, E2 becomes truncated and linear, and its expression is reduced. However, in our study, HPV16 integration sites were distributed across almost the whole genome, and cleavage was prone to occur in E1 (Figure 3b) [67]. These results were consistent with recent studies using NGS, including The Cancer Genome Atlas (TCGA) analysis of HPV-HNSCCs, that cleavage occurs in all regions, including E6, E7, and LCR, with the most truncation occurring in E1 [68,69,70,79,80]. It has been reported that the cleavage of E1 not only suppresses the function of downstream E2 but also induces local chromosomal instability at the integration site [81,82]. As another factor that can influence E2 expression, Reuschenbach et al. reported that the E2 binding site in the LCR of HPV-OPC specimens was classified into three groups based on the methylation rate and that in the hypermethylated group, the E2 binding affinity decreased and E6 and E7 were highly expressed. In addition, they reported that the hypermethylated group had the worst prognosis among the three groups [83]. Another factor is the involvement of apolipoprotein B messenger RNA editing, enzyme-catalytic, polypeptide-like 3 (APOBEC3), a cytidine deaminase that functions as an immune-defense mechanism to protect the host from foreign pathogens, such as viruses. In cervical intraepithelial neoplasia, it has been proposed that APOBEC3 causes mutations in the HPV genome and promotes carcinogenesis [84]. Kondo et al. reported that the expression of APOBEC3A mRNA increased in HPV-OPC samples and that G-to-A/C-to-T mutations were highly prevalent in E2 [85]. They proposed that these results, together with the report that HPV16 E6 and E7 induce APOBEC3A and 3 B expression [86], indicate that integrated HPV E6 and E7 induces APOBEC3 expression and may cause mutations in E2 [85]. Thus, various mechanisms other than E2 cleavage may be involved in the repression of E2 expression and the subsequent increase in the expression of E6 and E7 [87].

### 5.3. Viral-Host Sequences at the Integration Breakpoints

In our previous work on characterizing HPV integration, there were three distinct categories at the viral-host junctional sequences of the breakpoint: (1) seamless transition from one sequence to the next with a clearly defined breakpoint; (2) the presence of short inserted sequences at the breakpoint that match neither reference sequence; (3) breakpoint microhomology, defined as the presence of several bases of pairs of sequence homology at the breakpoint junction that could be assigned to either genome. In our samples, breakpoint microhomology accounted for the majority (Figure 3c). These results were consistent with those of other studies analyzing HPV integration breakpoints using NGS [68,79]. Gao et al. showed that the observation of these seamless transitions and short inserted sequences indicates that non-homologous end joining is the main mechanism underlying these rearrangements and that breakpoint microhomology contains AT-rich sequences that have the potential to form stem–loop structures that might stall DNA replication fork progression during replication stress [73]. Furthermore, microhomology-mediated break-induced replication may also be the causative mechanism of this rearrangement [79].

### 5.4. HPV Integration Types

HPV integration patterns have been classified into two types: type I, in which only one copy of HPV is integrated, and type II, in which multiple copies of HPV are integrated as tandem head-to-tail repeats [61]. Recently, Akagi et al. performed whole-genome sequencing and RNA sequencing (RNA-seq) analysis on HPV-SCC cell lines and clinical specimens to analyze the integration mechanisms and oncogenesis. They proposed the “looping model” to explain the events that give rise to viral-host concatemers from the formation of transient loops that serve as the substrate for rolling circle replication. In this process, focal amplification and rearrangement of the human genome and the HPV genome adjacent to the integration site occurs, resulting in a direct cause of chromosomal instability [69]. Furthermore, Warburton et al. proposed a type III integration in which tandem copies of the HPV genome interspersed with cellular DNA. They also showed that viral and host DNA could be co-amplified after the initial integration event [88].

### 5.5. Effect of HPV Integration on Cellular Genes

The accumulation of mutations resulting from integrating the HPV genome into the human genome is considered an important step in carcinogenesis. Parfenov et al. showed several mechanisms of HPV integration-induced gene disruption: loss of function of the tumor suppressor, overexpression of the oncogene by HPV integration upstream of the gene, amplification of the nearby downstream region, and interchromosomal translocation of chromosomes [68]. Gillison et al. showed using NGS analysis that several genes that were not expressed in HPV-negative cancers were upregulated in 149 HPV-positive OPC and oral-cancer samples. They indicated that HPV-positive and HPV-negative cancers may no longer be considered the same as OPC and that OPC should be classified according to the characteristics of host genetic mutations that interact with the viral genome [89].

### 5.6. HPV Integration Affects Epigenetic Alterations

We previously showed an association between the occurrence of integration and the methylation levels of the host genome at HPV integration sites. In addition, a significant correlation was observed between the methylation levels of HPV16 integrants and that of the flanking host genome. These findings suggest that HPV16 may adopt the methylation status of the flanking host genome in HPV-HNSCC, which may affect the expression of viral genes [67]. Furthermore, Warburton et al. proposed that when the integration site is in the vicinity of the host enhancer region and tandem repeats of the HPV LCR-host enhancer region occur, a novel Brd4-dependent super-enhancer-like effect is formed, which results in increased expression of genes derived from the HPV genome [62,88,90]. In these regions, the level of H3K27ac, a marker of active enhancer, is increased, indicating that chromatin is in an activated state. It has also been demonstrated that inhibiting the binding of Brd4 to acetylated histones by bromodomain and extra-terminal inhibitors causes a decrease in the expression of E6 and E7. These results suggest that the mechanism of HPV-associated carcinogenesis and integration is mediated not only by genomic rearrangements but also by epigenetic alterations.

## 6. HPV Protein-Host Protein Interactions

As an interesting study of the HPV protein, Eckhardt et al. reported an interactome analysis that comprehensively measures HPV protein-host protein interactions [91]. They indicated new interactions between E7-YAP1 and WNT/β-catenin signaling, which is known to be involved with E6 and E7, and E2 in addition to the previously shown involvement of Rb-E7 and p53-E6. Furthermore, by integrating this interactome analysis with gene expression analysis of genes that are mutated in HPV-negative samples but not in HPV-positive samples, they showed that several interactions that have not been previously linked to carcinogenesis, such as KEAP1-E1 and RNF20/40-L2 interactions, may be involved in carcinogenesis.

## 7. Concluding Remarks

In HPV-OPC, increased expression of E6 and E7 is important in oncogenesis; however, this may be one of several factors involved in the process. Considering that various factors associated with HPV-host interactions are responsible for genomic instability, no doubt escaping the host immune system and integrating into the host genome plays an essential role in HPV-associated oncogenic processes. However, many of these integrations are likely to be silent integrations owing to the increasing overall genetic instability. Most of these are considered to be passengers rather than drivers of carcinogenesis [62,92]. The evaluation of the mechanisms of HPV-associated carcinogenesis requires a more comprehensive functional analysis of the genome and epigenome of HPV and cancer immunity associated with HPV integration. Furthermore, spatial analysis involving not only tumor cells but also the tumor microenvironment and temporal changes are necessary for such analyses. Recent advances in molecular barcoding and single-cell RNA-seq technology have led to the development of analytical techniques, including spatial information at the single-cell level [93,94]. Wieland et al. investigated the humoral immune response in the tumor microenvironment of HPV-OPC. They showed that activated B cells, germinal center B cells, and HPV-specific antibody-secreting cells were present in the tumor microenvironment using single-cell RNA sequencing analyses [95]. A better understanding of the relationship between intratumor heterogeneity in cancer cells and the tumor microenvironment will facilitate the development of effective treatment strategies.

## Data Availability

Not applicable.
